# Muscle Tissue Damage and Recovery After EV71 Infection Correspond to Dynamic Macrophage Phenotypes

**DOI:** 10.3389/fimmu.2021.648184

**Published:** 2021-07-09

**Authors:** Mei-Yi Lu, Ya-Lin Lin, Yali Kuo, Chi-Fen Chuang, Jen-Ren Wang, Fang Liao

**Affiliations:** ^1^ Institute of Biomedical Sciences, Academia Sinica, Taipei, Taiwan; ^2^ National Institute of Infectious Diseases and Vaccinology, National Health Research Institutes, Tainan, Taiwan; ^3^ Department of Medical Laboratory Science and Biotechnology, National Cheng Kung University, Tainan, Taiwan; ^4^ Department of Pathology, National Cheng Kung University Hospital, Tainan, Taiwan; ^5^ Center of Infectious Disease and Signaling Research, National Cheng Kung University, Tainan, Taiwan

**Keywords:** EV71, enterovirus, myositis, skeletal muscle, calcification, macrophages

## Abstract

Enterovirus 71 (EV71) is a positive single-stranded RNA virus from the enterovirus genus of the *Picornaviridae* family. Most young children infected with EV71 develop mild symptoms of hand, foot and mouth disease, but some develop severe symptoms with neurological involvement. Limb paralysis from EV71 infection is presumed to arise mainly from dysfunction of motor neurons in the spinal cord. However, EV71 also targets and damages skeletal muscle, which may also contribute to the debilitating symptoms. In this study, we have delineated the impacts of EV71 infection on skeletal muscle using a mouse model. Mouse pups infected with EV71 developed limb paralysis, starting at day 3 post-infection and peaking at day 5-7 post-infection. At later times, mice recovered gradually but not completely. Notably, severe disease was associated with high levels of myositis accompanied by muscle calcification and persistent motor end plate abnormalities. Interestingly, macrophages exhibited a dynamic change in phenotype, with inflammatory macrophages (CD45^+^CD11b^+^Ly6C^hi^) appearing in the early stage of infection and anti-inflammatory/restorative macrophages (CD45^+^CD11b^+^Ly6C^low/-^) appearing in the late stage. The presence of inflammatory macrophages was associated with severe inflammation, while the restorative macrophages were associated with recovery. Altogether, we have demonstrated that EV71 infection causes myositis, muscle calcification and structural defects in motor end plates. Subsequent muscle regeneration is associated with a dynamic change in macrophage phenotype.

## Introduction

Enterovirus 71 (EV71) is a positive single-stranded RNA virus from the enterovirus genus of the *Picornaviridae* family (1). EV71 infection mainly affects young children. EV71 infection typically causes mild symptoms of hand, foot and mouth disease (HFMD) in young children (2), but it may also cause severe symptoms with neurological involvement and life-threating complications (3). One severe symptom that sometimes occurs in infants and young children infected with EV71 is acute flaccid paralysis (3, 4), which is thought to arise from spinal cord pathology that leads to motor deficits (5). However, it is also possible that damage to skeletal muscles may contribute to the debilitating symptoms. Since EV71 is a neurotropic virus, studies have mostly focused on the effects of EV71 on neuronal damage, and relatively few have probed the effects of EV71 on skeletal muscle. Recently, it was suggested that EV71 may spread from muscle to the CNS *via* retrograde axonal transport (6), and several mouse models have been used to show that EV71 is able to infect muscle cells (7, 8). Currently, the effects of EV71 on muscle tissue are rarely studied.

Hosts infected with muscle-tropic viruses may develop acute myositis with infiltrating leukocytes, resulting in muscle weakness (9). Since EV71 shows muscle tropism and infections sometimes result in acute flaccid paralysis (3–5), it is probable that EV71-induced muscle damage, in addition to EV71 effects on the CNS, could contribute to this paralysis. As such, virus-induced muscle weakness could result from the destruction of overall muscle structure and/or the integrity of motor end plates that form the postsynaptic portions of neuromuscular junctions (NMJs). Structural disruption of end plates would likely impair signal transmission from spinal cord lower motor neurons to the muscle. Interestingly, it has also been suggested that the NMJ could be a gateway for EV71 and other viruses to gain entry into neurons (10, 11). Thus, several potential effects of EV71 infection on muscle may be important mediators of disease.

The objective of this study was to assess the involvement of limb muscles in debilitating disease caused by EV71 infection. We found that mouse pups infected with EV71 developed severe myositis, muscle calcification, macrophage infiltration, and abnormal motor end plates in limb muscles during the early stage of EV71 infection. At later time-points EV71-induced damage in muscle tissue was repaired and restored, i.e., the inflammation, myositis and muscle calcification were gradually resolved, but motor end plates showed persistent abnormalities. Furthermore, we found that a dynamic change in macrophage phenotype was closely associated with muscle inflammation and regeneration phases of EV71 infection.

## Material and Methods

### Mice

C57BL/6 mice were originally purchased from the Jackson Laboratory and maintained in the specific pathogen free facility at the Institute of Biomedical Sciences, Academia Sinica (Taipei, Taiwan). All animal experiments were approved by the Institutional Animal Care and Utilization Committee (IACUC) at Academia Sinica and performed in accordance with institutional guidelines.

### Virus Infection

Mouse-adapted EV71 virus (12) was intraperitoneally injected into mouse pups (11-12 days old) at a dose of 8 × 10^5^ PFU per mouse. After viral infection, mice were monitored daily for clinical signs of limb paralysis and scored from 1 to 5: score 1, weakness in one or two hindlimbs (wobbling); score 2, paralysis in one hindlimb; score 3, paralysis in both hindlimbs; score 4, paralysis in both forelimbs and hindlimbs; score 5, death.

### Plaque Assay

The gastrocnemius and soleus muscles were collected from EV71-infected mice. Muscles were weighed and homogenized in PBS containing protease inhibitor cocktail (Merck). Muscle lysates were serially diluted in Dulbecco′s Modified Eagle′s Medium (DMEM) containing 2% fetal bovine serum (FBS) and added to a monolayer of RD (human rhabdomyosarcoma cell line; ATCC CCL-136) cells that were at a density of 5 × 10^5^ cells per well in 6-well plates. After 1 h incubation at 37°C, muscle lysates were removed, and RD cells were cultured in DMEM containing 2% FBS and 1% low melting agarose for 2 days. Cells were then fixed in 4% paraformaldehyde and stained with crystal violet solution. Viral plaques in each well were counted, and the plaque forming unit (PFU) was calculated.

### Total RNA Isolation and Real-Time Quantitative PCR

Gastrocnemius and soleus muscles were collected, homogenized in Trizol solution (Invitrogen) using homogenizer (Kinematica Polytron PT3100) and subjected to total RNA extraction according to the manufacturer’s instructions. Total RNA was treated with RQ1 RNase-free DNase (Promega) and was reverse transcribed into cDNA by SuperScript III Reverse Transcriptase (Invitrogen) according to the manufacturer’s instructions. The cDNA was subjected to quantitative PCR analysis on ABI QuantStudio^®^ 3 Real-Time PCR System (Applied Biosystems) using SYBR Green Master mix (Thermo Scientific). The thermal cycling protocol was 1 cycle at 50°C for 2 min and 95°C for 10 min, followed by 40 cycles of 95°C for 15 s and 60°C for 1 min. The resultant PCR products were analyzed with ABI QuantStudio Design & Analysis Software v.1.4.3 (Applied Biosystems). The relative levels of gene expression were calculated using the ΔCt method with normalization to *GAPDH*. DNA sequences of the primer pairs used in this study are listed in **Supplementary Table 1**.

### FACS Analysis

EV71-infected mice were anesthetized and perfused with PBS. Gastrocnemius and soleus muscles were collected, cut into small pieces and digested with 1.6 mg/ml collagenase IV (Sigma) and 330 µg/ml DNase I (BioShop) at 37°C for 30 min. After removal of tissue debris, cells were washed and resuspended in DMEM containing 10% FBS. For surface marker staining, cells were washed with FACS buffer (HBSS with Ca^2+^/Mg^2+^, 1% FBS, 10 mM HEPES and 0.1% NaN_3_) once, blocked with Fc blocker (clone 2.4G2, BD Pharmingen) on ice for 5-10 min, and then incubated with fluorochrome-conjugated antibodies against mouse CD45 (Brilliant Violet 785, clone: 30-F11, BioLegend), CD11b (PerCP/Cyanine5.5, clone: M1/70, BioLegend), Ly6C (Brilliant Violet 510, clone: HK1.4, BioLegend) and Ly6G (Brilliant Violet 650, clone: 1A8, BioLegend) on ice for 30 min. After two consecutive washes with HBSS, cells were stained with fixable viability dye (eFluor 780, eBioscience) on ice for 10 min. Cells were washed twice more and fixed in FACS buffer containing 0.8% paraformaldehyde. Intracellular staining was performed using a Fixation/Permeabilization solution kit (BD Biosciences), according to the manufacturer’s instructions. In short, cells were stained with antibodies to surface markers and viability dye, followed by fixation in Cytofix/Cytoperm solution (BD Biosciences). Cells were then washed twice with Fixation/Permeabilization solution (BD Biosciences) followed by incubation with normal rat serum at room temperature for 10 min. Next, cells were incubated with fluorochrome-conjugated antibody against mouse CD206 (Alexa Fluor 488, clone: C068C2, BioLegend) at room temperature for 30 min. Finally, cells were washed with Fixation/Permeabilization solution, resuspended in FACS buffer and subjected to FACS analysis. Cell populations were analyzed on a BD LSRII, and data were analyzed with FlowJo software (BD Biosciences).

### Immunofluorescence Staining

Mice were anesthetized by inhalation of isoflurane vapor. Proper anesthetization was confirmed by a lack of deep tendon reflex. The gastrocnemius muscle was excised and fixed with 4% paraformaldehyde in PBS at 4°C for 24 h, followed by PBS washing. The muscle was embedded in OCT (Sakura Finetek) and longitudinally sectioned using a cryostat (Leica CM3050). For immunostaining, the muscle sections (10 µm) were incubated with blocking buffer (0.1% Triton X-100, 1% BSA, 10% normal goat serum in PBS) at room temperature for 1 h. The muscle sections were then incubated with primary antibodies in antibody diluent (0.1% Triton X-100, 1% BSA, 2% normal goat serum in PBS) overnight at 4°C. After washing in PBS for 30 min, the sections were incubated with secondary antibodies in the antibody diluent at room temperature for 2 h. After washing in PBS for 30 min, the muscle sections were counterstained with DAPI, mounted and observed under confocal microscopy (LSM800 with Airyscan, ZEISS or LSM700 stage, ZEISS). Antibodies used for immunofluorescence staining and other procedures are listed in **Supplementary Table 2**.

To analyze the structures of NMJs in gastrocnemius muscle, tissue sections (50 µm) were incubated with blocking buffer (0.5% Triton X-100, 1% BSA, 10% normal goat serum in PBS) at room temperature for 2 h, followed by incubation with acetylcholine receptor (AChR) binding toxin, tetramethylrhodamine α-bungarotoxin (α-BTX, BIOTIUM), at room temperature for 2 h. After washing in PBS for 30 min, muscle sections were counterstained with DAPI, mounted and observed under confocal microscopy (LSM800 with Airyscan, ZEISS or LSM700 stage, ZEISS). Z-stack images of AChRs were obtained and illustrated with maximum intensity projection.

### H&E Staining and Alizarin Red S Staining

Muscles excised from mice were fixed in 4% paraformaldehyde/PBS at 4°C for 24 h, then dehydrated, embedded in paraffin and transversely sectioned at 5 µm. Sections were subjected to H&E staining for histological analysis or Alizarin Red S staining for muscle calcification. Images were scanned at 40× magnification using Pannoramic 250 Flash II (3D HISTECH Ltd.) and analyzed with Pannoramic Viewer software and ImageJ software (NIH Image). The image acquisition and analysis are shown on **Supplementary Figure 1**.

### Statistical Analysis

The non-parametric one-way ANOVA (Kruskal-Wallis test) with Dunn’s multiple comparison test was used to evaluate the significant differences on different infection time points. *P-*values < 0.05 were considered statistically significant. Statistical analysis was performed using GraphPad Prism software version 8.

## Results

### Mouse Pups Infected With EV71 Exhibit Rapid Induction of Inflammatory Cytokines, Chemokines and Anti-Viral Molecules in Skeletal Muscle

**Figure 1:**
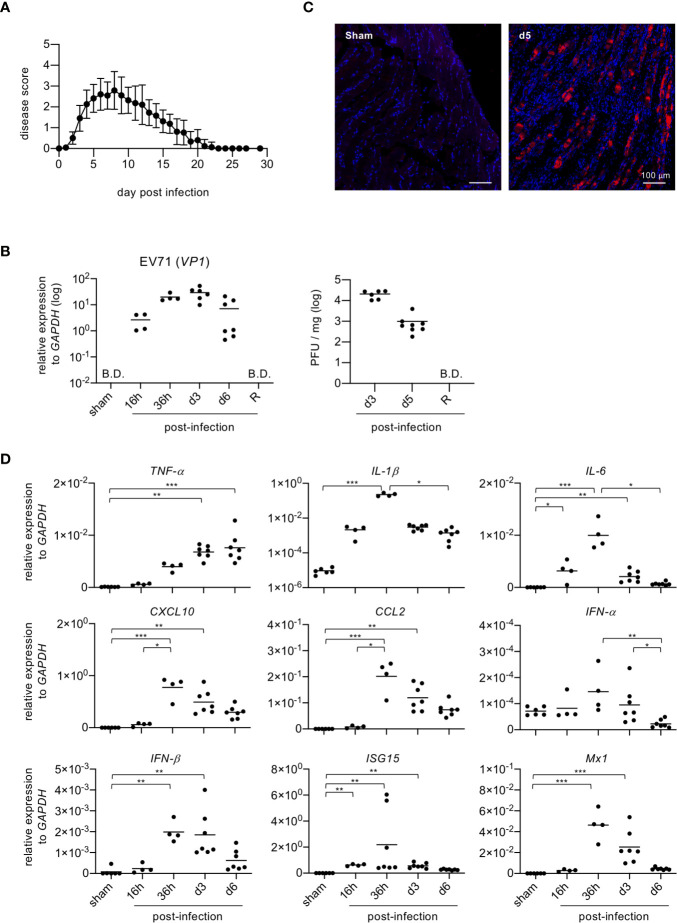
Mouse pups infected with EV71 show rapid induction of inflammatory cytokines and anti-viral molecules in skeletal muscle. **(A)** Mouse pups (P11-P12, n = 23) were injected i.p. with EV71 virus (8 × 10^5^ PFU) at day 0 and monitored daily for limb paralysis. Clinical scores: 0, no sign of disease; 1, weakness in one or two hindlimbs (wobbling); 2, paralysis in one hindlimb; 3, paralysis in both hindlimbs; 4, paralysis in both forelimbs plus both hindlimbs; 5, death. **(B)** Gastrocnemius and soleus muscles were collected from sham-infected or EV71-infected mice at indicated times, and real-time quantitative PCR was performed to determine gene expression levels of EV71 viral gene *vp1* (left panel). Lysates from gastrocnemius and soleus muscles of EV71-infected mice at day 3 and day 5 post-infection, and at recovery phase (R) were subjected to plaque assay (right panel). R represents mice that recovered from limb paralysis and wobbly status (score = 1). Each symbol represents one mouse (n = 4-8). B.D. represents below determination. **(C)** Cryosections of gastrocnemius muscle from sham-infected or EV71-infected mice at day 5 post-infection were subjected to immunofluorescence staining with the rabbit antibody to viral protein VP1 and then Cy3-conjugated anti-rabbit IgG (red) followed by DAPI counterstain (blue). **(D)** Real-time quantitative PCR was used to probe gene expression levels of pro-inflammatory cytokines and chemokines (*TNF-a, IL-1β, IL-6, CXCL10* and *CCL2*) and anti-viral molecules (*IFN-α, IFN-β, ISG15* and *Mx1*) in muscle samples described for [**(B)**, left panel]. Gene expression levels were normalized to *GAPDH. P* values in **(B, D)** were calculated by Kruskal-Wallis test with Dunn’s multiple comparison test. *P < 0.05; **P < 0.01; ***P < 0.001; ****P < 0.0001.

In severe cases, EV71 infection may cause limb paralysis in infants and young children (2). The EV71-induced limb paralysis is thought to result from damage to the CNS; however, the effects of EV71 on skeletal muscle are largely unknown. Thus, we examined the effect of EV71 infection on skeletal muscle in a mouse model. As with humans, adult mice are resistant to EV71 infection, and only mouse pups are susceptible to its effects. Mouse pups (11-12 days old) were infected with EV71 (8 × 10^5^ PFU), and limb paralysis was assessed with a clinical scoring system. We noted that mouse pups infected with EV71 exhibited exceptionally stiff limb muscles, which is more likely due to a musculoskeletal defect than neurological dysfunction (13, 14). The onset of disease was about 2-3 days post-infection (dpi), and the peak of disease was about 5-7 dpi. Mice then recovered from limb paralysis at about 20 dpi or later (**Figure 1A**). Analysis of virus titers in limb muscles (gastrocnemius and soleus muscles) revealed that virus loads peaked at 3 dpi and declined rapidly after 6 dpi and no virus was detected in the recovery phase (**Figure 1B**). We then examined whether EV71 targets muscle cells by performing immunofluorescence staining of muscle tissue sections with an antibody against viral protein, VP1, revealing that the viral protein could be readily detected in muscle tissues (**Figure 1C**). Since the viral load declined rapidly at 6 dpi, we speculated that muscle cells might induce anti-viral molecules to clear virus. We therefore examined the expression of anti-viral molecules after EV71 infection. Muscle tissues were collected from limbs at various times post-infection, and quantitative PCR was performed to measure the gene expression of pro-inflammatory cytokines, chemokines and anti-viral molecules. Several pro-inflammatory cytokines and chemokines (*TNF-α*, *IL-1β*, *IL-6*, *CXCL10, CCL2*) as well as anti-viral molecules (*IFN-α*, *IFN-β*, *ISG15*, *Mx1*) were induced upon EV71 infection. Notably, these molecules except *TNF-α* were induced as early as 16 hours post-infection, peaked at around 36 hours post-infection, and significantly declined at 6 dpi (**Figure 1D**). These results demonstrate that EV71 infects skeletal muscles and induces pro-inflammatory cytokines, chemokines and anti-viral molecules that are expected to promote viral clearance.

### Mouse Pups Infected With EV71 Show Severe Myositis Accompanied by Calcification

**Figure 2:**
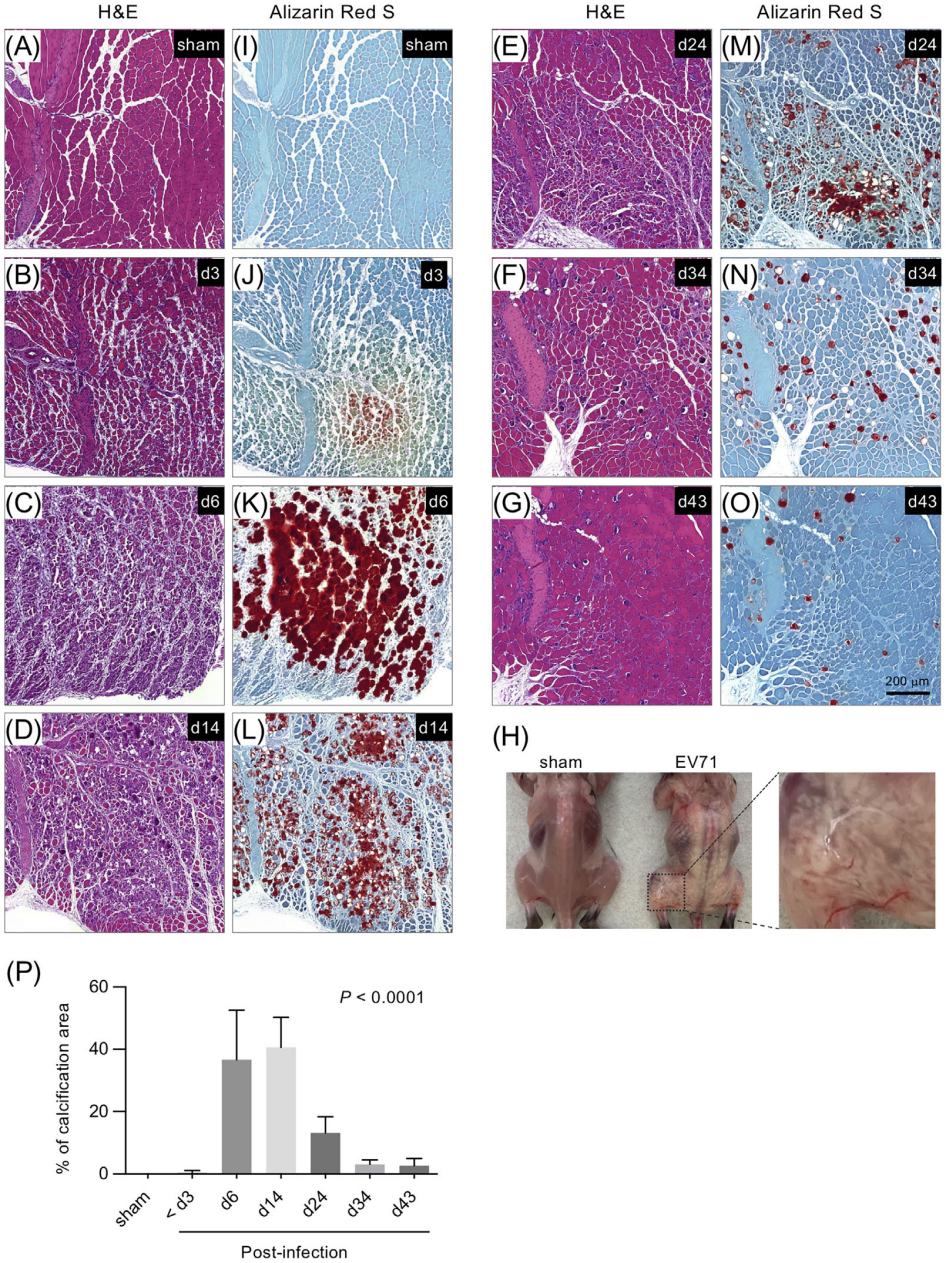
Histopathological staining of the gastrocnemius muscle from EV71-infected mice. Gastrocnemius muscle sections from sham-infected or EV71-infected mice at day 3 (score = 1), day 6 (score = 3.5), day 14 (score = 1), day 24 (score = 0.5), day 34 (score = 0), and day 43 (score = 0) were stained with H&E staining **(A-G)** or Alizarin Red S staining **(I-O)**. **(H)** Photos of representative sham-infected and EV71-infected mice at day 8 post-infection (score = 3) are shown. The EV71-infected mouse (d8) showed a white appearance in muscles of body trunk and limbs. The magnified image highlights the aberrant structure of muscles with white appearance. **(P)** The percentage of calcification areas of muscles were measured and calculated as mentioned in **Supplementary Figure 1**. The number of mice used for the quantification of image and their paralysis scores were indicated below: sham-infected (n = 2) or EV71-infected mice at day 3 (n = 3, score = 0, 0.5-1), day 6 (n = 2, score = 3.5), day 14 (n = 4, score = 0.5~1), day 24 (n = 3. score = 0.5~1.5), day 34 (n = 2, score = 0) and day 43 (n = 2, score = 0) post-infection. *P* value was calculated using one-way ANOVA. *P* < 0.0001.

We next examined whether EV71 infection impaired muscle tissue structure, which might weaken the muscle and, at least in part, contribute to the loss of limb function and limb paralysis. Histopathological staining of gastrocnemius muscle sections revealed that EV71 infection resulted in severe myositis, which peaked at 5-7 dpi (**Figure 2** and **Supplementary Figure 2**). The muscle structure showed clear pathology, including leukocyte infiltration at 3 dpi (**Figure 2B**), and severe myositis with highly disrupted muscle fibers accompanied by severe leukocyte infiltration was observed at 5-7 dpi (**Figure 2C**). The myositis and infiltrating leukocytes gradually waned over the subsequent weeks, and muscle tissues were largely restored (**Figures 2D–G**). Of note, mouse pups infected with EV71 showed extensive abnormal white coloration in muscles of the body trunk and limbs (**Figure 2H**). Like the myositis, the white appearance also peaked at 6-7 dpi and then gradually disappeared. We next used Alizarin Red S staining to examine whether limb muscles with white appearance exhibited calcification; this stain is used to identify calcium deposits in tissue sections. The results revealed that the white appearance in the muscle tissues corresponded to positive Alizarin Red S staining, suggesting that the white appearance in the muscle tissue reflects calcification (**Figures 2J–O**). The percentage of calcification arears of muscles were calculated, which peaked at 6-7 dpi and then gradually waned (**Figure 2P**). This calcification of muscles may explain the stiffness of limb muscles we observed. Additionally, the severe myositis was concurrently observed with extensive calcification in muscles (**Figure 2**), and the muscle inflammation and calcification gradually diminished in parallel as the muscle structure recovered (**Figure 2** and **Supplementary Figure 2**). Taken together, these results suggest that EV71 infection induces severe myositis correspondent with muscle calcification, which destroys muscle structure and may underlie the loss of limb function.

### Different Macrophage Phenotypes Dynamically Appear During the Progression of EV71-Induced Myositis and Muscle Regeneration

**Figure 3:**
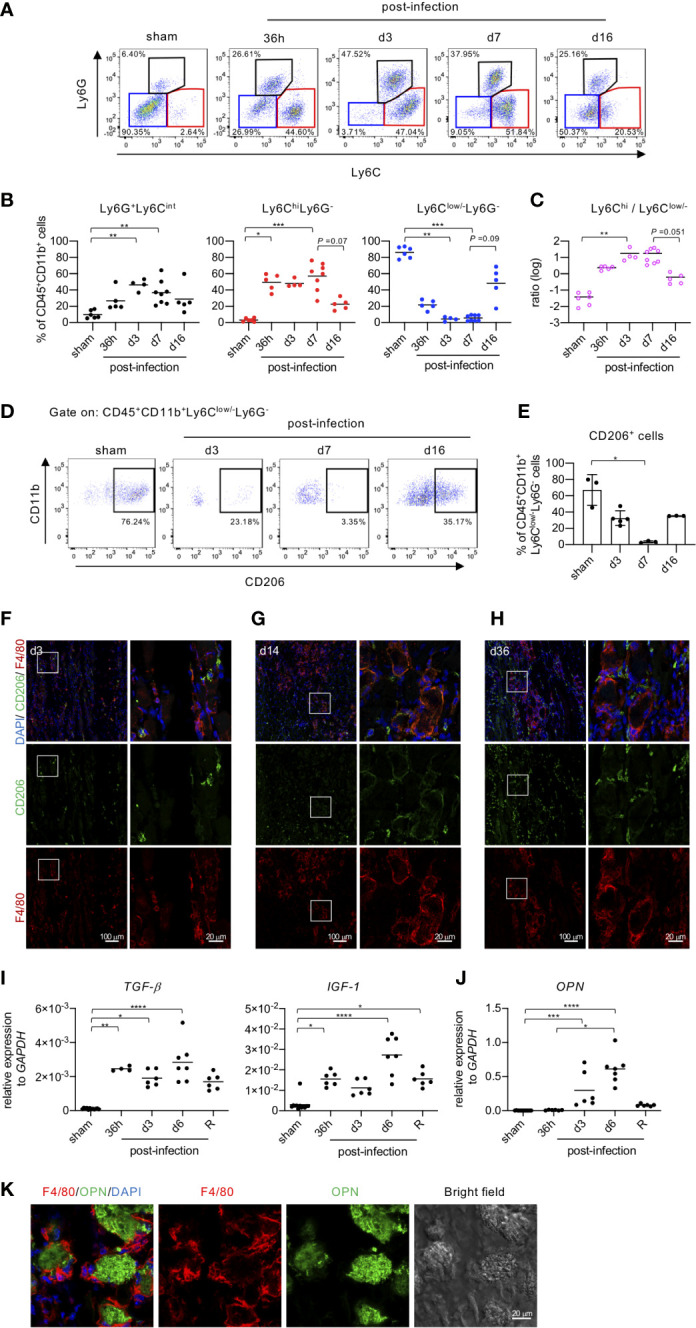
Macrophage phenotypes change in accordance with EV71-induced myositis and muscle regeneration. **(A-C)** FACS analysis of myeloid populations in muscle tissues. EV71-infected mice were sacrificed on the indicated day post-infection. Cells isolated from hindlimb muscles (gastrocnemius and soleus) were subjected to surface staining for CD45, CD11b, Ly6C, Ly6G and intracellular staining for CD206. Live single muscle cells were first gated on myeloid cells (CD45^+^CD11b^+^) followed by further gating on neutrophils (Ly6G^+^Ly6C^int^), monocytes/inflammatory macrophages (Ly6C^+^Ly6G^-^) and anti-inflammatory/restorative macrophages (Ly6C^low/-^Ly6G^-^). Representative pseudo-color plots are shown **(A)**. The frequencies of myeloid populations in muscles of EV71-infected mice are shown **(B)**. The ratio of inflammatory macrophages to anti-inflammatory/restorative macrophages is shown **(C)**. Each symbol represents one mouse. **(D, E)** FACS analysis of CD206 expression in anti-inflammatory/restorative macrophages (CD45^+^CD11b^+^Ly6C^low/-^Ly6G^-^). Representative pseudo-color plots **(D)** and the frequencies **(E)** are shown. Each symbol represents one mouse (n = 3-5). **(F-H)** Cryosections of gastrocnemius muscle in representative EV71-infected mice at day 3 (score = 2) **(F)**, day 14 (score = 1) **(G)**, and day 36 (score = 0) **(H)** post-infection were immunostained for CD206 (green) and F4/80 (red). The indicated regions from the left panels are shown magnified in the right panels. The relative gene expression levels of *TGF-β, IGF-1* **(I)** and *OPN* **(J)** in gastrocnemius and soleus muscle of EV71-infected mice were analyzed by real-time quantitative PCR and normalized to *GAPDH*. Each symbol represents one mouse (n = 4-8). R represents recovery. **(K)** Sections of gastrocnemius muscle from EV71-infected mice at day 14 post-infection were stained with OPN (green) and F4/80 (red). Images were obtained by LSM700 or LSM880 confocal microscopy. *P* values in **(B, C, I, J)** were calculated by Kruskal-Wallis test with Dunn’s multiple comparison test. *P < 0.05; **P < 0.01; ***P < 0.001; ****P < 0.0001.

Although mice infected with EV71 developed severe myositis associated with extensive calcification at the peak of disease (5-7 dpi), the pathology gradually resolved as time went on (**Figure 2**). We found this dynamic change in pathology of muscle tissues from severe myositis to regeneration of myofibers after EV71 infection to be quite intriguing, so we looked at other aspects of the tissue during this period. The major population of infiltrating leukocytes in muscle tissues with severe myositis appeared to be monocytes/macrophages, as shown by H&E staining (**Figures 2B–D**). We thus analyzed myeloid cell populations (CD45^+^CD11b^+^) in muscle tissues at various times post-infection using FACS analysis. The CD45^+^CD11b^+^ cells were identified as the myeloid cell population and were subjected to further analysis of Ly6G and Ly6C expression (**Figure 3A**). The Ly6G^+^Ly6C^int^ cells represented neutrophils (**Figure 3B**, left panel), while CD45^+^CD11b^+^Ly6C^hi^ and CD45^+^CD11b^+^Ly6C^low/-^ were considered to be monocytes/macrophages since they were all positive for F4/80 (data not shown). Furthermore, Ly6C^hi^Ly6G^-^ cells were considered to be inflammatory macrophages, and Ly6C^low/-^Ly6G^-^ cells were considered anti-inflammatory/restorative macrophages (15–18). The kinetics of neutrophil infiltration showed a peak at 3 dpi, followed by a gradual decline (**Figure 3B**, left panel). Ly6C^hi^ macrophages are expected to be recruited from circulating monocytes to the tissue during tissue damage (19), and accordingly, the percentage of Ly6C^hi^ macrophages was significantly increased in muscle tissue as early as 36 hours post-infection. The high levels were sustained until 7 dpi and declined at 16 dpi (**Figure 3B**, middle panel). On the other hand, Ly6C^low/-^ macrophages are typically resident tissue macrophages and/or anti-inflammatory/restorative macrophages, which function to resolve inflammation and promote regeneration of damaged tissues (15–18). The frequency of Ly6C^low/-^ macrophages was reduced upon EV71 infection as early as 36 hours post-infection, and it was further reduced at 3 and 7 dpi; the level was restored at 16 dpi (**Figure 3B**, right panel). These results show that macrophages with different phenotypes appear at different times in the process of EV71 infection and resolution, as indicated by the ratio of Ly6C^hi^/Ly6C^low/-^ macrophages (**Figure 3C**). The kinetics of Ly6C^hi^ and Ly6C^low/-^ macrophages in the muscle during EV71 infection were reciprocal, i.e., the increased frequency of Ly6C^hi^ macrophages (inflammatory macrophages) was accompanied by a decreased frequency of Ly6C^low/-^ macrophages (anti-inflammatory/restorative macrophages) during the early stage of EV71 infection, and a decreased frequency of Ly6C^hi^ macrophages was accompanied by an increased frequency of Ly6C^low/-^ macrophages during late stage of recovery from EV71 infection.

CD206, the mannose receptor, is a marker for tissue-resident and anti-inflammatory macrophages (20), and it is widely used to identify macrophage subsets that exhibit anti-inflammatory functions (21). Before infection, the major population of monocytes/macrophages was Ly6C^low/-^ macrophages, which also exhibited high expression of CD206 (**Figures 3A, D, E**). The frequency of CD206^+^ cells was gradually reduced upon EV71 infection, and it reached the lowest level at the peak of disease (7 dpi) with a rebound at the recovery phase (16 dpi) (**Figures 3D, E**). IFA staining revealed that at 3 dpi, damaged muscles were infiltrated with F4/80 macrophages, which were presumably inflammatory macrophages recruited to promote clearance of virus and cell debris. Some F4/80 macrophages were co-stained with different levels of CD206 (**Figure 3F**). At the later stage of infection, we observed macrophages around the periphery of damaged muscle fibers (**Figures 3G, H**), likely contributing to the removal of debris from tissues. This time corresponded to the resolution of inflammation and calcification as well as the regeneration of the muscle tissue. Of note, IFA staining showed that CD206^+^/F4/80^-^ cells increased with time in muscle tissues after EV71 infection (**Figures 3F–H**). These cells might be muscle satellite cells since CD206 is known to be expressed in that cell type (22). These results confirm that anti-inflammatory/restorative macrophages increase at the late stage of infection (**Figures 3A, B**) and suggest that anti-inflammatory/restorative macrophages may play an important role in repairing EV71-induced muscle damage and promoting regeneration (**Figures 3G, H**).

Since we suspected that anti-inflammatory/restorative macrophages might play a role in repairing tissue damage and regenerating muscles, we next examined gene expression for molecules produced by macrophages and known to be involved in tissue regeneration. The gene expression levels of *TGF-β* and *IGF-1*, which are produced by anti-inflammatory/restorative macrophages and promote repair and regeneration of damaged tissues (18, 23–25), were significantly increased at 36 h post-infection to at least 6 dpi and then declined in the recovery phase (**Figure 3I**). The gene expression level of osteopontin (OPN), which is known to regulate the regeneration of muscles following injury (26) and inhibit ectopic calcification (27), was also significantly increased at 3 dpi and further increased at 6 dpi and declined in the recovery phase (**Figure 3J**). We then further investigated the role of OPN in EV71-induced muscle calcification by immunofluorescence staining. We found that OPN was localized within the calcified area of muscle, likely to inhibit more calcification (**Figure 3K**); notably, macrophages surrounded the calcified area in the muscle tissue (**Figure 3K**). Altogether, the results from **Figure 3** suggest that macrophages with Ly6C^hi^ in the muscle tissue at the early stage of EV71 infection are inflammatory macrophages responsible for the initiation of inflammation to clear virus and debris, while macrophages in the muscle tissue with Ly6C^low/-^ appear at the late stage of EV71 infection and are anti-inflammatory/restorative macrophages that facilitate the resolution of inflammation and calcification as well as the regeneration of muscles.

### EV71 Infection Induces Impaired Postsynaptic Structure of the Neuromuscular Junction

**Figure 4:**
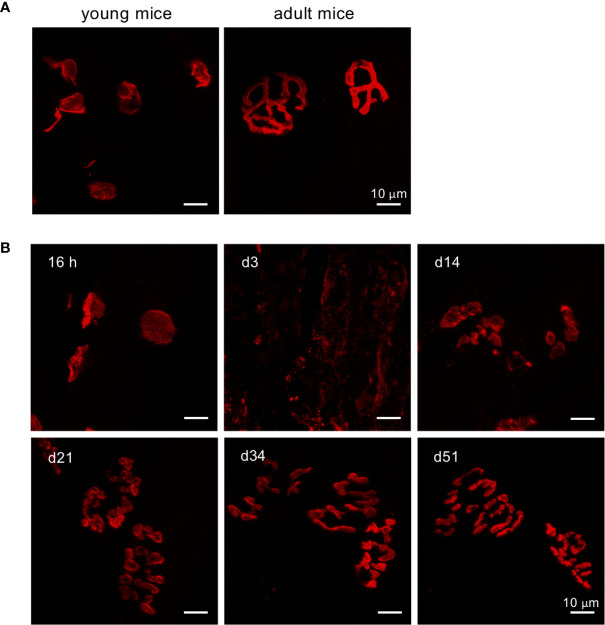
Defects in postsynaptic NMJ structure in the gastrocnemius muscle of EV71-infected mice. **(A, B)** Cryosections of gastrocnemius muscle from sham-infected young mice (P15), sham-infected adult (P34) **(A)**, or EV71-infected mice at 16 h (score = 0), day 3 (score = 2), day 14 (score = 1), day 21 (score = 0.5), day 34 (score = 0.5), and day 51 (score = 0) post-infection **(B)** were stained with tetramethylrhodamine α-BTX and observed under LSM700 confocal microscopy.

NMJs are specialized synapses between muscles and motor neurons, which control muscle movement. Given that EV71 infection induces weakness of limb muscles, severe myositis and muscle calcification, we rationalized that the NMJs in muscles may also be dysfunctional. Thus, we examined the structure of postsynaptic motor end plates in the muscle, specifically in the gastrocnemius muscle, by staining acetylcholine receptors (AChRs) in the postsynaptic membrane of the muscle fiber. It has been reported that the shapes of postsynaptic AChR clusters in young mice (2-3 weeks old) are quite different from those in adult mice. In mice, the shape of postsynaptic AChR clusters in NMJs is expected to change from plaque-like in young animals to pretzel-like in adult mice (28, 29). We identified innervated synapses by staining tissue sections of gastrocnemius muscle with α-bungarotoxin (α-BTX), which binds AChR (the marker for postsynaptic motor end plates). As expected, the structures of motor end plates in the muscle were plaque-like (**Figure 4A**, left panel) in tissues from uninfected young mice (15 days old), while a pretzel-like structure (**Figure 4A**, right panel) was observed in muscles from uninfected adult mice (34 days old). In virus treated mice, the structure of motor end plates from the muscle seemed to be unaffected at 16 hours post-infection, but the end plates became aberrant/fragmented at 3 dpi, when compared with uninfected 15-day-old mice. The motor end plates were gradually repaired and became more cohesive as time went on; the degree of repaired motor end plate structures was greatest at 51 dpi and progressively lower at 34, 21 and 14 dpi (**Figure 4B**). The motor end plates in infected mice did not appear to recover the pretzel-like structures seen in uninfected adult mice, even at 51 dpi (**Figure 4B**, d51 vs. **Figure 4A**, right panel). The motor end plate structures seemed repaired macroscopically at day 51 dpi. However, when counting fragments within a AChR cluster, we found that almost all AChR clusters even at 51 dpi (**Supplementary Figure 3**) showed five or more fragments per AChR cluster, which has been considered as an aberrant/fragmented AChR cluster (30). Of interest, the macroscopical structure of AChR clusters seemed improved along with the time, but the microscopical structure of an AChR cluster (aberrant/fragmented AChR cluster) seemed to stay impaired (**Supplementary Figure 3**) even at 51 dpi. These results suggest that EV71 infection might drastically impair postsynaptic motor end plates of NMJs, resulting in incompletely innervated synapses. This structural damage would inevitably affect the transmission of signals from lower motor neurons to the limb muscle, leading to the loss of limb function.

### Limb Muscles Impaired by EV71 Infection Are Able to Undergo Muscle Regeneration

**Figure 5:**
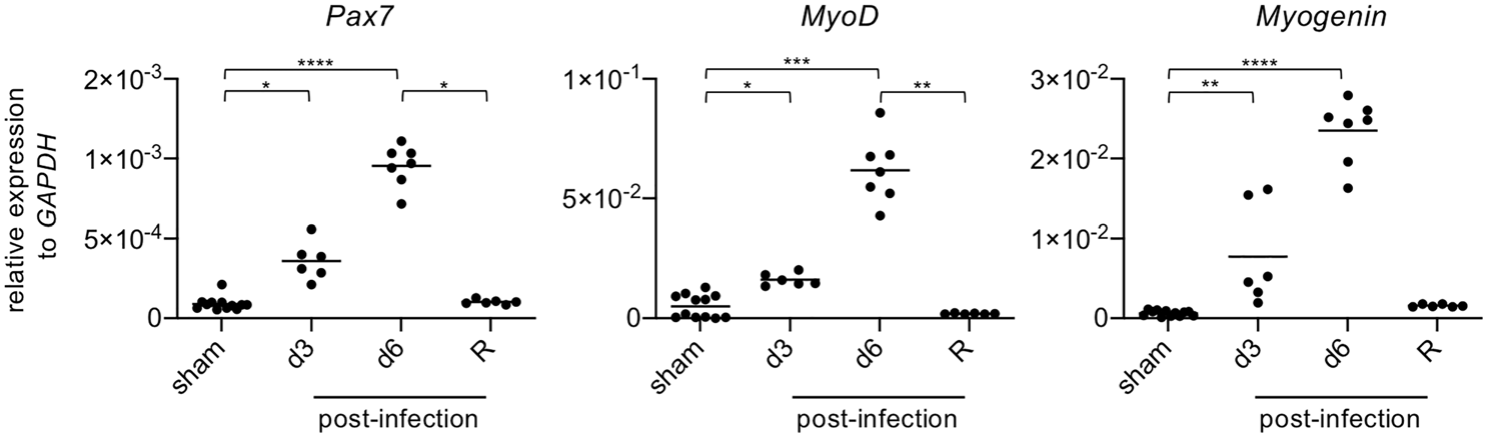
The levels of gene expression of muscle-related transcription factors are increased in damaged muscles after EV71 infection. The gastrocnemius and soleus muscles of sham-infected (P14, n = 6; P29, n = 6) or EV71-infected mice at day 3 and day 6 post-infection, and at the recovery phase were collected (day 15-23 post-infection) and subjected to real-time quantitative PCR analysis to determine gene expression levels of *Pax7*, *MyoD*, and *Myogenin*. The relative gene expression levels were normalized to *GAPDH*. Each symbol represents one mouse (n = 6-12). *P* values were calculated by Kruskal-Wallis test with Dunn’s multiple comparison test. *P < 0.05; **P < 0.01; ***P < 0.001; ****P < 0.0001.

The aforementioned results show that at late stage of infection, anti-inflammatory/restorative macrophages (Ly6C^low/-^) are increased (**Figures 3B, C**), while molecules responsible for tissue repair (*TGF-β*, *IGF-1*, *OPN*) are upregulated (**Figures 3I, J**), and the postsynaptic motor end plates of NMJs are repaired (**Figure 4B**). Based on these results, it appeared that limb muscles damaged by EV71 infection underwent regeneration. Pax7 is a transcription factor expressed in muscle satellite cells that regulates cell survival, proliferation and differentiation (31). Moreover, MyoD and Myogenin are transcription factors involved in skeletal muscle development, myogenesis and repair (32, 33). Analyzing gene expression levels by quantitative PCR, we found that *MyoD*, *Myogenin* and *Pax7* in limb muscles were all significantly increased at 3 dpi, further increased at 6 dpi, and declined in the recovery phase (**Figure 5**). Taken together, these results suggest that limb muscles damaged by EV71 infection are indeed able to undergo muscle regeneration. However, whether EV71-infected mice can completely regenerate damaged muscles remains unclear since the muscle morphologies at days 51 post-infection were still not completely normal (**Figure 4B**).

## Discussion

Since EV71 is known as a neurotropic virus, its effects on limb paralysis have been investigated almost entirely in terms of damage to the CNS (8, 12, 34). Whether weakness or damage to limb muscles also contributes to limb paralysis has not been previously shown. In this study, we investigated the effects of EV71 infection on the limb muscle. We demonstrated that EV71 infection induces severe myositis, which is gradually resolved and followed by restoration of the limb muscle. Furthermore, EV71 infection results in severe defects in motor end plates, the postsynaptic structures of NMJs. Interestingly, the end plates failed to completely recover, even months after infection. The defective motor end plates are likely to have a negative impact on the transmission of signals from lower motor neurons in the ventral horn of the spinal cord to the limb muscle, which may impair muscle function and exacerbate paralysis.

The infection of muscle cells with EV71 induced the production of pro-inflammatory cytokines, chemokines and anti-viral molecules at a very early time-point (16 hours post-infection), and this effect peaked at 36 hours post-infection (**Figure 1D**). Subsequently, myositis became evident, peaking at 5-7 dpi (**Figure 2C**). The early induction of innate molecules in EV71-infected muscle tissues is probably critical for inducing inflammation that promotes virus clearance, despite the accompanying severe myositis. Importantly, we found that the severe myositis was associated with large areas of white appearance, which we identified as sites of muscle calcification (**Figure 2K**). Our finding that EV71 infection induces muscle damage with large areas of white appearance (**Figure 2H**) seems similar to a previous report, which showed that EV71 infection induces “white-jade” patches in muscles (7). Thus, our results and the previous study, by Liu *et al.*, independently confirmed a similar phenomenon, which may be specific to EV71 infection. Liu *et al.* further showed that the “white-jade” phenomenon is a result of hypoxia; we extended this finding by showing that the phenomenon is attributable to muscle calcification. The next question for future study will naturally be whether hypoxia is associated with calcification in muscle tissues infected by EV71. Several reports have demonstrated that cellular hypoxia indeed triggers/promotes heterotopic calcification (35, 36). Additionally, previous studies have shown that hypoxia-inducible factor-1 (HIF-1) is a regulator of bone morphogenic protein 2 (BMP-2)-induced endochondral ossification in fetal limb culture (37) and that HIF-1-transduced bone marrow stem cells undergo osteogenic differentiation (38). It is therefore plausible that EV71 infection results in hypoxia-induced calcification of limb muscle tissues, and, interestingly, the hypoxia-induced calcification appears not to be restricted to the limb muscles (**Figure 2H**).

Upon muscle injury, muscle tissue damage and subsequent repair processes are always accompanied by the dynamic engagement of molecules from infected cells and infiltrating leukocytes. In particular, macrophages have been shown to play an important role in the process of muscle injury (18), as infiltrating monocytes transition from inflammatory macrophages (Ly6C^hi^) to anti-inflammatory/reparative macrophages (Ly6C^low/-^) required for muscle regeneration (18, 39, 40). Similar to the observation in muscle injury, we found that the majority of infiltrating leukocytes in muscle tissues during EV71 infection were macrophages and that inflammatory macrophages (Ly6C^hi^) appeared at the early stage of viral infection, while anti-inflammatory/reparative macrophages (Ly6C^low/-^) appeared at the late stage of viral infection. These results suggest that upon EV71 infection of muscle tissues, Ly6C^hi^ macrophages are important for the regulation of inflammation, while Ly6C^low/-^ macrophages are critical for the resolution of inflammation and regeneration of muscles. Inflammatory macrophages (Ly6C^hi^) are thought to be circulating monocytes recruited to muscle tissues *via* the CCR2/CCL2 axis, where they produce pro-inflammatory cytokines and chemokines in concert with infected muscle cells that produce anti-viral molecules to restrict viral replication. These processes occur at the expense of tissue inflammation and tissue damage. In addition, it has been reported that cardiotoxin injury-induced myositis is critical for the subsequent accumulation of anti-inflammatory/restorative macrophages (Ly6C^low/-^) that stimulate repair of damaged tissues (18, 39). Macrophages scattered throughout uninfected tissues are Ly6C^low/-^ tissue-resident macrophages and also show high expression of CD206. Notably, anti-inflammatory/restorative macrophages (Ly6C^low/-^) also express CD206 (**Figures 3D, E**). After infection, the frequency of Ly6C^low/-^ macrophages typically declines gradually, while the frequency of Ly6C^hi^ macrophages gradually increases during the early stage of infection (up to about 3 dpi). Later, the frequency of anti-inflammatory/restorative macrophages (Ly6C^low/-^) is gradually recovered, while the frequency of inflammatory macrophages (Ly6C^hi^) declines at late stage of infection (**Figure 3**). In line with the increased frequency of anti-inflammatory/restorative macrophages (Ly6C^low/-^) during the late stage of infection, we observed increased gene expression of several molecules, especially *TGF-β*, *IGF-1* and *OPN*, which are known to be produced by anti-inflammatory/restorative macrophages and are associated with the resolution of inflammation, inhibition of calcification, and regeneration of muscles (18, 23, 24, 26) (**Figures 3I, J**). Intriguingly, our immunofluorescence staining revealed that macrophages stained with CD206 and F4/80 (presumably anti-inflammatory/restorative macrophages) displayed an elongated morphology and surrounded damaged and/or calcified muscle fibers (**Figures 3G, H, K**). This morphology and localization suggest that the anti-inflammatory/restorative macrophages may be promoting the resolution of inflammation, inhibiting calcification and facilitating the regeneration of damaged muscles. Moreover, the dynamic change in macrophage phenotypes revealed a reciprocal relationship between the frequency of Ly6C^hi^ macrophages and Ly6C^low/-^ macrophages (**Figures 3B, C**), suggesting that anti-inflammatory/restorative macrophages might be derived from inflammatory macrophages in response to changes in the microenvironmental cues present during the progression of EV71 infection and resolution. The signals regulating interchangeable macrophage phenotypes remain to be investigated. Of interest, our results on dynamic macrophages phenotypes changes in EV71-induced myositis is reminiscent of the recent publication, which showed that myositis induced by Ross River virus (an Arthritogenic alphaviruses) infection is driven by CD11b^hi^Ly6C^hi^ inflammatory monocytes followed by the establishment of CD11b^hi^Ly6C^low/-^ macrophages for the recovery of damaged muscles (41). It is plausible that, during viral infection, the muscle tissue damage and subsequent repair seem associated with the differentially producing various cytokines/soluble factors to regulate the macrophage phenotype for eradicating and resolving infection. These observations might be a physiological phenomenon observed in myositis induced by virus infection, which is worthy of further investigation.

Based on our immunofluorescence data (**Figures 3G, H, K**), EV71-induced muscle damage was always accompanied by muscle calcification (**Figure 2**), and damaged areas were surrounded by macrophages (**Figures 3G, H**), suggesting that macrophages may take part in the inhibition of calcification and the regeneration of muscles. Given that it is important to resolve muscle calcification prior to muscle regeneration and that OPN is reported to be secreted by macrophages (27), one may expect that muscle calcification would be tightly associated with OPN (**Figure 3K**), which inhibits calcification. We did observe increased levels of OPN at the late stage of infection (**Figures 3J, K**). Based on these facts, it is plausible that during the late stage of viral infection, damaged muscles become calcified and are surrounded by macrophages, which produce OPN to inhibit muscle calcification prior to muscle regeneration.

We also found that the motor end plates in the muscle, the postsynaptic structure of NMJs, were significantly affected by EV71 infection (**Figure 4B**). The disruption of motor end plate morphology by EV71 infection may be attributable to the aberrant muscle structure and/or absence of an appropriate cellular milieu for proper maturation of NMJs. Surprisingly, the motor end plates in infected mice were not fully repaired to resemble those in uninfected adult mice, even at 51 dpi (**Figure 4**). Despite the continued aberrant morphology of end plates, mice still appeared to walk without noticeable difficulty. It is possible that subtle movement defects exist, however, we did not perform substantive examinations of gait or other features of motility. Since the motor end plates are required for transmission of electrical impulses from the motor neurons to the skeletal muscle that stimulate muscle contraction, motor end plates with aberrant structure are likely to have functional defects that result in the loss of muscle contractility and/or weakness of the muscle. Our recent work has demonstrated that EV71 targets motor neurons in the ventral horn of the spinal cord, leading to limb paralysis (42). The muscular defects induced by EV71 infection shown in the present study are expected to worsen the limb paralysis induced by spinal cord motor neuron dysfunction after EV71 infection.

In sum, we have demonstrated that EV71 infection results in deteriorating muscle tissue, marked by inflammation and calcification, as well as aberrant motor end plates at the early stage of infection, and later, resolution of the severe myositis and calcification is coincident with increased presence of anti-inflammatory/restorative macrophages in the tissue, resulting in regenerating muscle tissues. Importantly, we demonstrated that macrophage phenotypes are dynamic in the process of EV71 infection, suggesting that the cells play different roles at different times. Inflammatory macrophages (Ly6C^hi^) should induce inflammation at early stages, while anti-inflammatory/restorative macrophages (Ly6C^low/-^) may resolve inflammation to promote regeneration of muscles.

## Data Availability Statement

The original contributions presented in the study are included in the article/**Supplementary Material**. Further inquiries can be directed to the corresponding author.

## Ethics Statement

All animal experiments were approved by the Institutional Animal Care and Utilization Committee at Academia Sinica, Taipei, Taiwan (IACUC protocol # 11-12-264).

## Author Contributions

M-YL and Y-LL designed and performed experiments, and analyzed data. C-FC and YK performed experiments and analyzed data. J-RW provided a crucial virus clone. FL conceived and designed the study, supervised experiments, analyzed data and wrote the manuscript. All authors contributed to the article and approved the submitted version.

## Funding

This study was supported by grants from the National Science Council in Taiwan (NSC99-2321-B-001-035, NSC100-2321-B-001-025, NSC101-2321-B-001-013) and from the Institute of Biomedical Sciences, Academia Sinica, Taipei, Taiwan.

## Conflict of Interest

The authors declare that the research was conducted in the absence of any commercial or financial relationships that could be construed as a potential conflict of interest.
