# Supplementary material for: Muscle Tissue Damage and Recovery After EV71 Infection Correspond to Dynamic Macrophage Phenotypes
Source: Front Immunol. 2021 Jul 9;12:648184. doi: 10.3389/fimmu.2021.648184 (PMC8299204; doi:10.3389/fimmu.2021.648184)
Supplement: Supplementary file 1 [file DataSheet_1.pdf]

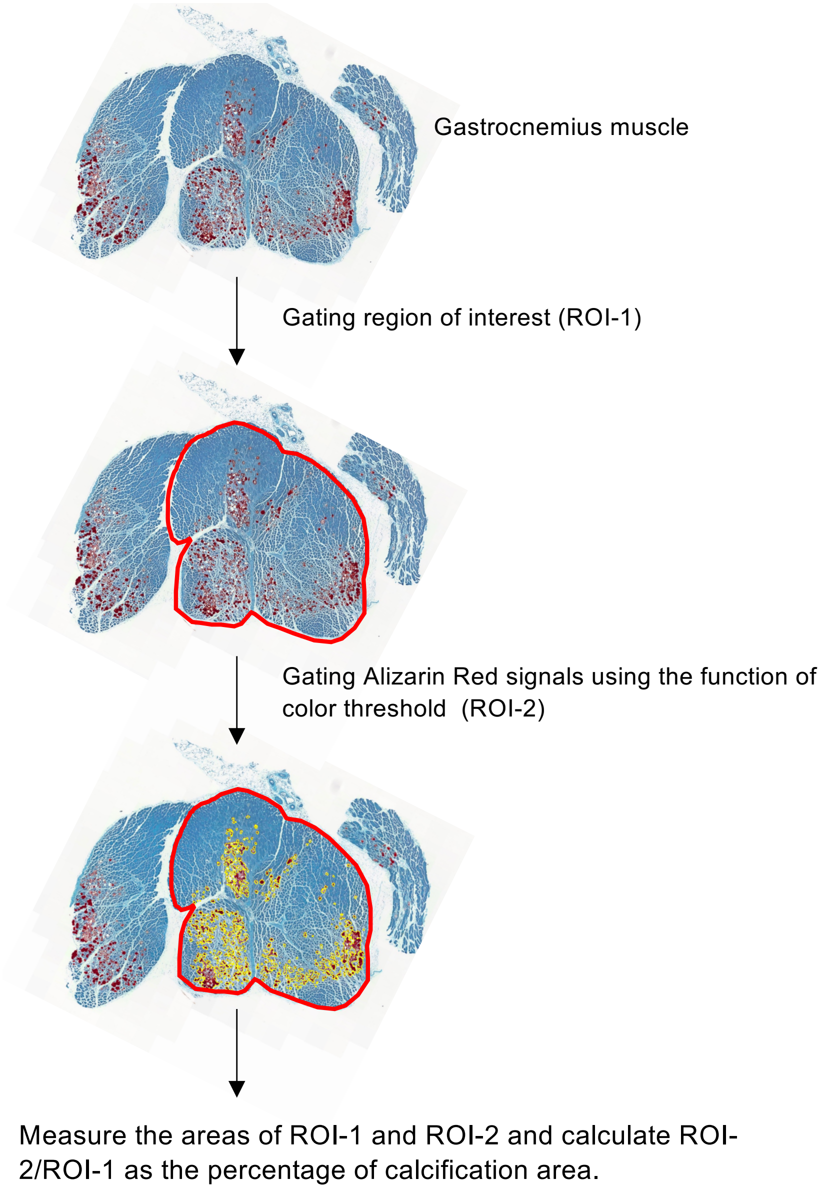


**Supplementary Figure 1. The histological image acquisition and analysis in gastrocnemius muscles.**

The gastrocnemius muscle of the left leg was collected, fixed and sectioned. The region of interest (ROI-1) was selected (red-circled area), which region was most close to the infection site and showed good tissue integrity. The function of image adjustment with the color threshold was used to measure the Alizarin Red signals (ROI-2, yellow area), representing the levels of calcification. The areas of ROI-1 and ROI-2 were quantified, and the ratio of ROI-2/ROI-1 was calculated as the percentage of calcification area.

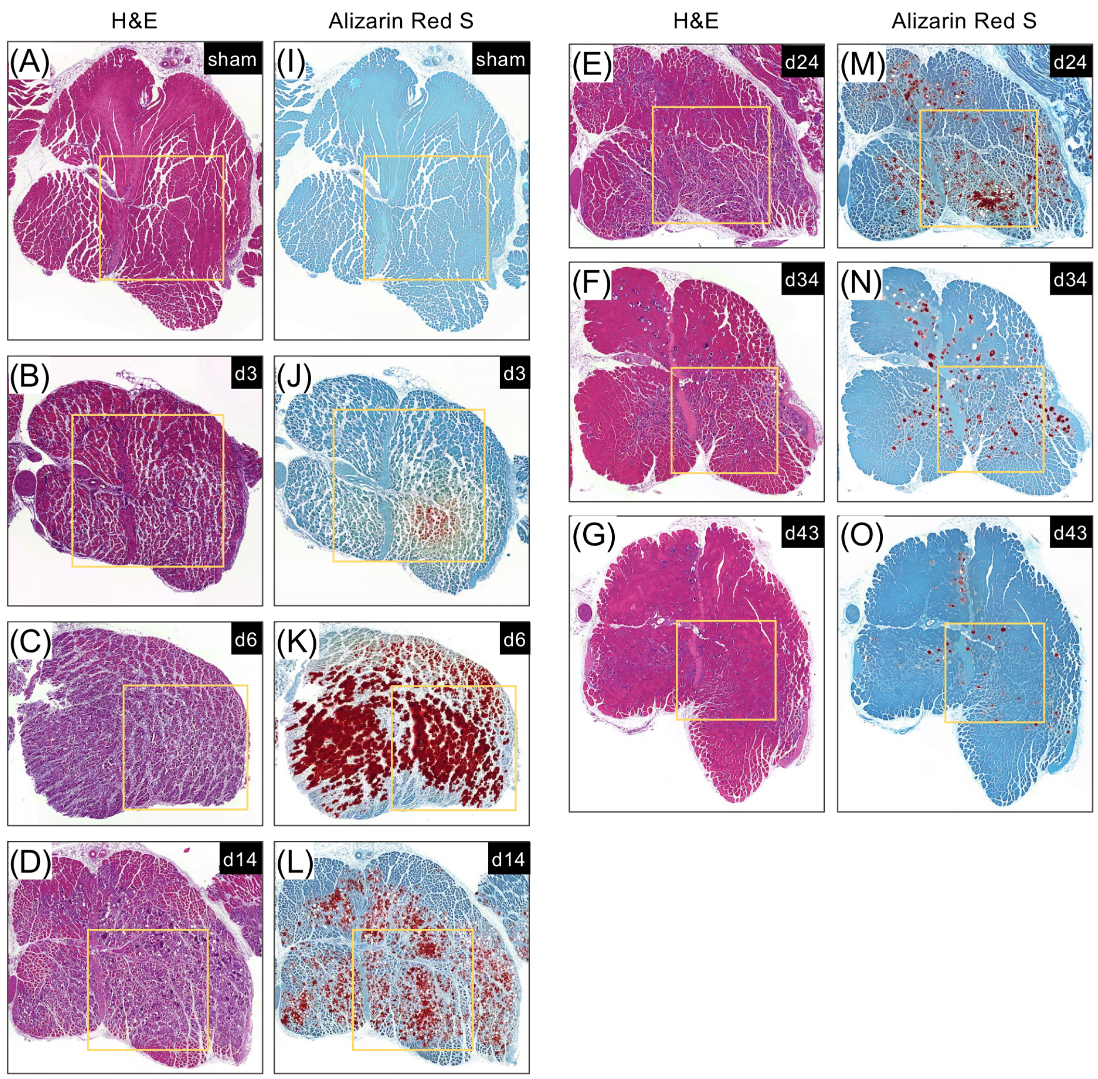


**Supplementary Figure 2. The histological staining of the gastrocnemius muscle from sham-infected and EV71-infected mice.**

The representative images of H&E staining (A-G) and Alizarin Red S staining (I-O) of gastrocnemius muscle sections from sham-infected or EV71-infected mice at day 3 (score = 1), day 6 (score = 3.5), day 14 (score = 1), day 24 (score = 0.5), day 34 (score = 0) and day 43 (score = 0) post-infection were shown. The square areas were magnified and presented as the Figure 2 in the manuscript. The scale of each square is 1 mm × 1 mm.

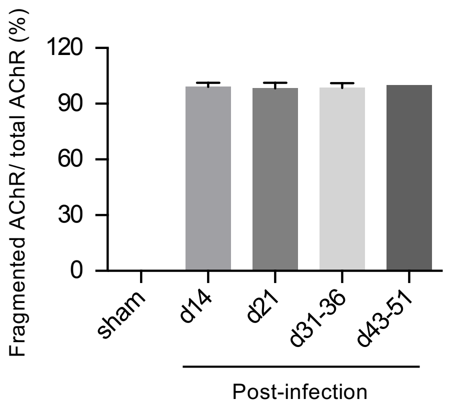


**Supplementary Figure 3. The quantification of fragmented AChR clusters in the gastrocnemius muscles of sham-infected and EV71-infected mice.**
The Z-stack images of gastrocnemius muscle sections stained with tetramethylrhodamine α-BTX were obtained and illustrated with maximum intensity projection. The number of discrete fragments per AChR cluster equals to or more than 5 was defined as a fragmented AChR cluster (30). The percentage of fragmented AChR clusters was measured and calculated from sham-infected (Total 44 AChR clusters from 2 mice were counted) or EV71-infected mice at day 14 (65 AChR clusters from 5 mice), day 21 (59 AChR clusters from 3 mice), day 31-36 (48 AChR clusters from 3 mice) and day 43-51 (35 AChR clusters from 2 mice) post-infection.

**Supplementary Table 1.** Primer sequences used for quantitative PCR.

| Murine gene | Forward | Reverse |
|---|---|---|
| *VP1* | GTGGCAGATGTGATTGAGAG | GTTATGTCTATGTCCCAGTT |
| *IRF7* | TCCAGCGAGTGCTGTTTGGA | CGAGCCTCGTTCAGCCA |
| *TNFα* | TTCTCATTCCTGCTTGTGGCA | TGATGAGAGGGAGGCCATTTG |
| *IL-1β* | TGTAATGAAAGACGGCACACC | TCTTCTTTGGGTATTGCTTGG |
| *IL-6* | GATGGATGCTACCAAACTGGAT | CCAGGTAGCTATGGTACTCCAGA |
| *IFN-α* | ARSYTGTSTGATGCARCAGGT | GGWACACAGTGATCCTGTGG |
| *IFN-β* | AGGGCGGACTTCAAGATC | CTCATTCCACCCAGTGCT |
| *ISG15* | ACTCCATGACGGTGTCAGAAC | TTCGTTCCTCACCAGGATGC |
| *Mx1* | CCAACTGGAATCCTCCTGGAAAA | CCTTCTCCTCATAGTGCCTGC |
| *CXCL10* | TGAGCAGAGATGTCTGAATC | TCGCACCTCCACATAGCTTACAG |
| *CCL2* | CAGTTAACGCCCCACTCACC | ATTCCTTCTTGGGGTCAGCA |
| *TGF-b* | AAGGCTCGCCAGTCCCCCAA | TAGATGGCGTTGTTGCGGTCCAC |
| *IGF-1* | ACCTCTTCCCACGTAGCTCA | TGCCACAGATGGAGTCAGGT |
| *OPN* | GATGATGATGACGATGGAGACC | CGACTGTAGGGACGATTGGAG |
| *Pax7* | AGGCCTTCGAGAGGACCCAC | CTGAACCAGACCTGGACGCG |
| *MyoD* | CAGCATCACGGTGGAGGATA | CAGTTGGGCATGGTTTCGT |
| *Myogenin* | GGCTGCCTAAAGTGGAGATCCT | AGGCCTGTAGGCGCTCAAT |
| *GAPDH* | TACTTGGCAGGTTTCTCCAG | GTCGTGGAGTCTACTGGTGT |

**Supplementary Table 2**. Antibodies used in immunofluorescence staining and staining procedures.

(A) Staining for F4/80 and CD206

| Step | Antibody | Manufacturer |
|---|---|---|
| 1 | Rat anti-F4/80 (clone: BM8) | BioLegend |
| 2 | Alexa 555 goat anti-rat IgG | Molecular Probes |
| 3 | Alexa 488 rat anti-CD206 (clone: C068C2) | BioLegend |

(B) Staining for EV71 VP1

| Step | Antibody | Manufacturer |
|---|---|---|
| 1 | Rabbit anti-EV71 VP1 | GeneTex |
| 2 | Cy3 goat anti-rabbit IgG | Molecular Probes |
